# Autism spectrum traits in normal individuals: a preliminary VBM analysis

**DOI:** 10.3389/fnhum.2015.00264

**Published:** 2015-05-12

**Authors:** Farah Focquaert, Sven Vanneste

**Affiliations:** ^1^Department of Philosophy and Moral Sciences, Bioethics Institute Ghent, Ghent UniversityGhent, Belgium; ^2^Lab for Clinical and Integrative Neuroscience, School of Behavioral and Brain Sciences, Callier Center for Communication Disorders, The University of Texas at DallasDallas, TX, USA

**Keywords:** VBM, autism spectrum disorders, systemizing, empathizing

## Abstract

In light of the new DSM-5 autism spectrum disorders diagnosis in which the autism spectrum reflects a group of neurodevelopmental disorders existing on a continuum from mild to severe expression of autistic traits, and recent empirical findings showing a continuous distribution of autistic traits in the general population, our voxel based morphometry study compares normal individuals with high autistic traits to normal individuals with low autistic traits. We hypothesize that normal individuals with high autistic traits in terms of empathizing and systemizing [high systemizing (HS)/low empathizing (LE)] share brain irregularities with individuals that fall within the clinical autism spectrum disorder. We find differences in several social brain network areas between our groups. Specifically, we find increased gray matter (GM) volume in the orbitofrontal cortex, the cuneus, the hippocampus and parahippocampus and reduced GM volume in the inferior temporal cortex, the insula, and the amygdala in our HS/LE individuals relative to our HE/LS (low autistic traits in terms of empathizing and systemizing) individuals.

## Introduction

According to defenders of the continuum view (e.g., Widiger and Trull, 2007; Livesly, 2012), the endophenotypes that give rise to a given psychiatric disorder should be understood as extreme instances of *normal* cognitive-emotional and/or personality traits that exist along a continuum. The empirical evidence supports a dimensional view involving continuous types with a cut-off point being used to convert a continuous distribution into a diagnosis, rather than a categorical view involving discontinuous types (Widiger, 2011; Livesly, 2012). Although the new DSM-5 continues to separate normal from abnormal traits in a discontinuous way, the new autism spectrum disorders (ASDs) diagnosis does reflect a group of neurodevelopmental disorders that exist on a continuum from mild to severe expression, involving impairments in the social-communicative domain (e.g., deficits in social-emotional reciprocity) and behavioral domain (e.g., fixated interests and repetitive behaviors). The DSM-5 ASD diagnosis no longer separates autism from Asperger syndrome (involving autistic traits and preserved cognitive functioning) due to consistent findings of overlap between the diagnostic criteria (Lauritsen, 2013). Moreover, accumulating evidence places autistic traits on a continuum in the general population, with clinical ASD representing the extreme end of this continuous distribution (Whitehouse et al., 2011). Baron-Cohen (2008) describes autism spectrum conditions as empathizing–systemizing conditions, in which individuals with autism spectrum conditions show below average empathizing alongside normal or above average systemizing. The empathizing–systemizing theory of psychological sex differences (Baron-Cohen, 2003; Baron-Cohen et al., 2005) claims that whereas the female brain is predominantly hard-wired for empathy, the male brain is predominantly hard-wired for understanding and building systems. Empathizing can be defined as “the drive to identify another’s mental states and to respond to these with an appropriate emotion, in order to predict and to respond to the behavior of another person" (Baron-Cohen et al., 2005, p. 820). Unless an individual has a pathologically high level of empathy, individuals with high empathizing skills typically feel comfortable in social settings, can easily relate to others and are able to form close, lasting relationships with others. These individuals are very socially sensitive. In contrast, individuals with low empathizing (LE) typically feel uncomfortable during social settings, have difficulty understanding others’ thoughts and feelings, and often find it difficult to establish and maintain social relationships. Women typically score higher on empathy (as measured by the Empathy Quotient questionnaire or EQ) compared to men (Baron-Cohen, 2003). Systemizing can be defined as “the drive to analyze a system in terms of the rules that govern the system, in order to predict the behavior of the system" (Baron-Cohen et al., 2005, p. 820). Individuals that score high on systemizing are good at understanding mechanical systems and input–output relations in general. They have the ability to focus on relevant details and ignore irrelevant details, and are often engineers or scientists. Individuals with low systemizing skills are typically less good at understanding mechanical systems, input–output relations. Men typically score higher on systemizing (as measured by the Systemizing Quotient questionnaire or SQ) compared to women (Baron-Cohen, 2003). According to the E–S theory individuals can possess three particular ‘brain types’: (1) an individual’s level of empathy can be higher than his or her level of systemizing (E > S), (2) an individual’s level of systemizing can be higher than his or her level of empathizing (S > E) or (3) an individual can have comparable levels of empathizing and systemizing skills (S = E). The second type (S > E) is more common in men, whereas the first type (E > S) is more common in women (Baron-Cohen et al., 2005; Wright and Skagerberg, 2012). Individuals with extreme E > S cognitive patterns are deemed ‘system-blind,’ whereas individuals with extreme S > E cognitive patterns are deemed ‘mind-blind.’ The ‘extreme male brain theory’ (EMB) holds that autism represents an extreme of the male brain type (S > > E; Baron-Cohen et al., 2005). According to the EMB individuals with ASD are characterized by impairments in empathizing while having intact or superior systemizing skills. Several studies provide supporting evidence for the empathizing-systemizing theory of psychological sex differences and the related EMB theory (Baron-Cohen et al., 2003; Lawson et al., 2004; Wakabayashi et al., 2007; Aiello and Whitaker-Azmitia, 2011; Brosnan et al., 2012). For example, Wheelwright et al. (2006) examined the EMB in a large UK sample that compared students (723 males, 1038 females) to adults with ASD (69 males, 56 females) and found that the majority (62%) of adults with ASD had an extreme male brain type (S > > E), compared to only 5% of typical males and 0.9% of typical females. Wakabayashi et al. (2007) found similar results in a Japanese sample showing cross-cultural stability of the different empathizing–systemizing ‘brain types’ and further supporting the empathizing–systemizing theory of psychological sex differences and the related EMB theory.

The EMB theory is in line with a dimensional view involving continuous types since empathizing and systemizing skills are cognitive-emotional traits that exist along a continuum in the normal population. Empathizing can be defined as “the drive to identify another’s mental states and to respond to these with an appropriate emotion, in order to predict and to respond to the behavior of another person” (Baron-Cohen et al., 2005, p. 820). Systemizing can be defined as “the drive to analyze a system in terms of the rules that govern the system, in order to predict the behavior of the system” (Baron-Cohen et al., 2005, p. 820).

Recently, several meta-analyses found white and gray matter (GM) structural abnormalities in individuals with ASD compared to controls in the lateral occipital and temporo-occipital region, the superior pericentral region (i.e., precentral, central, and postcentral gyrus), the precuneus, the region of the superior insula and adjacent parietal operculum, the anterior cingulate, the middle temporal gyrus, the hippocampus, the amygdala, the basal ganglia, and the cerebellum (Stanfield et al., 2008; Cauda et al., 2011; Yu et al., 2011; Nickl-Jockschat et al., 2012). Moreover, Ecker et al. (2013) found cortical thickness and cortical volume differences in individuals with ASD, mainly located in frontal and temporal regions, that correlated significantly with symptom severity. Several of the GM irregularities found in ASD are located within the social brain network underlying social abilities such as mindreading and empathy. The social brain network comprises areas such as the temporal cortex, orbitofrontal cortex, the medial prefrontal cortex, the insula, and the amygdala (Baron-Cohen, 1995; Pelphrey et al., 2011).

If individuals with ASD do indeed possess ‘extreme male brains’ in terms of empathizing and systemizing, then (some of the) the structural brain abnormalities that are found in these individuals might also be found (to a lesser extent) in normal individuals with ASD traits in terms of empathizing and systemizing [i.e., individuals with high systemizing (HS) and LE]. The goal of our study is to examine whether *normal* individuals with ASD traits in terms of empathizing and systemizing (HS/LE) share certain brain irregularities with individuals that fall within the clinical ASD group. The dimensional view of psychiatric disorders and the ‘EMB of autism’ both predict at least some *shared* brain abnormalities (or irregularities) in individuals with ASD and normal individuals with ASD traits. We therefore hypothesize that the comparison of the HS/LE to the HE/LS (i.e., high/above average empathizing and low systemizing) individuals will reveal a pattern of brain differences in the HS/LE individuals relative to the HE/LS individuals that overlaps with structural brain abnormalities in individuals diagnosed with ASD. More specifically, we hypothesize that the HS/LE and HE/LS individuals will show GM differences in the occipital lobe, the temporo-occipital region, the pericentral region, the temporal lobe (the fusiform gyrus and parahippocampal gyrus), the basal ganglia (the caudate nucleus, the putamen), the insula/operculum, the hippocampus, the amygdala, the precuneus, and/or the cerebellum analog to previous findings within the ASD literature.

## Materials and Methods

### Selection of Participants

#### Questionnaires

##### Empathizing Quotient (EQ)

The empathizing quotient (Baron-Cohen and Wheelwright, 2004) is a validated 60-item (40 assessing empathy and 20 filler control) measure assessing empathy. Responses are given on a four-point-Likert scale ranging from strongly disagree to strongly agree. Responses were scored in the suggested manner, with participants receiving zero for a ‘non-empathic’ response, one for a somewhat empathic and two for a very empathic response (e.g., strongly agree gets two points, agree gets one point, and disagree and strongly disagree both get zero points; or strongly disagree gets two points, disagree gets one point, and agree and strongly agree both get zero points). The total possible score is 80.

##### Systemizing Quotient (SQ)

The systemizing quotient (Baron-Cohen et al., 2003) is a validated 60-item (40 assessing systemizing and 20 filler control) measure assessing systemizing. Responses are given on a four-point-Likert scale ranging from strongly disagree to strongly agree. Responses were scored in the suggested manner, with participants receiving zero for a ‘non-systemizing’ response, one for a somewhat systemizing response, and two for a very systemizing response (e.g., strongly agree gets two points, agree gets one point, and disagree and strongly disagree both get zero points; or strongly disagree gets two points, disagree gets one point, and agree and strongly agree both get zero points). The total possible score is 80.

#### Participants Included

One-hundred-and-thirty-seven volunteers were recruited from the Dartmouth student and *post doc* pool in mathematics, physics, chemistry, computer science, engineering, environmental science, theater, etc. Participants were screened using the empathy quotient and systemizing quotient (Baron-Cohen, 2003). A score between 0 and 32 on the empathizing quotient is a low score (most people with Asperger Syndrome or high-functioning autism score about 20), between 33 and 52 is average (most women score about 47, and most men score about 42), between 53 and 63 is above average and between 64 and 80 is very high (80 is the maximum). A score between 0 and 18 on the systemizing quotient is low, between 20 and 39 is average (with most women scoring about 24 and most men scoring about 30), between 40 and 50 is above average (most people with Asperger Syndrome or high-functioning autism score in this range), and between 51 and 80 is very high (three times as many people with Asperger Syndrome score in this range, as compared to typical men and almost no women score in this range, 80 is the maximum; Baron-Cohen, 2003). Out of the total group of participants (*n* = 137), we selected a subgroup of 24 participants (mean age = 27.29 years, range 18–37 years) along the empathizing quotient (mean age = 27.58 years, range 23–30 years) and systemizing quotient (mean age = 27 years, range 18–38 years) results style (see **Table 1**). One half of the subgroup included 12 participants with HS and LE and constitutes the systemizing group or HS/LE individuals, and the other half of the subgroup included 12 participants with above average empathizing (most men score about 42) and below average systemizing (most men score about 30) and constitutes the empathizing group or HE/LS individuals. The HS/LE group scored, on average, within the Asperger range on both systemizing quotient and empathizing quotient, whereas the HE/LS group scored, on average, higher than most normal men on the empathizing quotient, and lower than most men on the systemizing quotient. All individuals in the HS/LE group had to score a minimum of 40 on the systemizing quotient to be included (i.e., in the Asperger range). We then further selected those individuals with the lowest matching empathizing quotient scores. The individuals in the HE/LS group had the highest scores on the empathizing quotient, with a minimum score of 33 as a cut-off point, matched with the lowest scores on the systemizing quotient, with a maximum score on the systemizing quotient of 39 as a cut-off point. Thus, their empathizing quotient score was as high as possible within the average to high range, and their matching systemizing quotient was as low as possible within the average or low range. In sum, although the HE/LS individuals scored within the normal ranges of the population as a group, they averaged toward a more female-typical pattern and were the most female-typical scoring men within the entire pool in terms of systemizing quotient/empathizing quotient scores (i.e., normal men with low autistic traits). Participants were paid $10 per hour for their participation in the behavioral part and informed consent was obtained in accordance with the guidelines set forth by the Dartmouth Committee for the Protection of Human Subjects (CHPS protocol #17772).

**Table 1:** Descriptive statistics.

		*N*	Minimum	Maximum	Mean	SD
e HS/LE group	SQ	12	40.00	59.00	47.83	6.08
= SQ > EQ	EQ	12	12.00	43.00	30.83	8.42
HE/LS Group	SQ	12	10.00	38.00	27.92	8.91
= EQ > SQ	EQ	12	36.00	70.00	47.00	11.86
**Systemizing group – Empathizing group**
	ΔSQ		30.00	21.00	19.91	−2.83
	ΔEQ		−24.00	−27	−16.17	−3.44

Most individuals with Asperger syndrome or high-functioning autism score within the ‘above average’ range on the systemizing quotient scale (i.e., 40–50 out of 80), and within the ‘low’ range on the empathizing quotient scale (i.e., 0–32 out of 80), typically having a score of about 20 out of 80 on the latter. The individuals in our systemizing group scored within the Asperger range on both systemizing quotient and empathizing quotient, whereas the individuals in our empathizing group scored, on average, lower than most normal men on the systemizing quotient, and higher than most normal men on the empathizing quotient. Hence, our systemizing group displayed an Asperger-type pattern, i.e., HS combined with LE, without ever been diagnosed with ASD. On the systemizing quotient, most normal men score about 30 out of 80, whereas on the empathizing quotient most normal men score about 42 out of 80 (for ranges see Baron-Cohen, 2003). All participants were male to ensure that sex differences in brain structure did not confound our findings (Lentini et al., 2013; Ingalhalikar et al., 2014). All participants were right-handed, had normal visual acuity, and were screened for history of psychiatric or medical illness. Exclusion criteria for all participants included history of depression, anxiety, bipolar disorder, eating disorder, Asperger syndrome, sleep disorder, and alcoholism. The entire pool of participants (*n* = 137) was asked to indicate if they had ever been diagnosed with any of these disorders by answering yes or no in writing to each individual item on a list. To ensure honest disclosure, the lists did not include biographical information. Each participant list received an ID number that was linked to their biographical information in a separate document to ensure strict confidentiality. This was mentioned in writing on top of each list, and participants were asked to answer honestly. Only participants answering no to each item on the list are included in the imaging study.We did not screen first degree relatives.

### Voxel Based Morphometry

#### Image Acquisition

Imaging was performed on a three Tesla Phillips Scanner using a SENSE head coil for signal reception. 3D-high resolution sagittal images were collected for each subject using the following parameters: TR = 9.9 ms, TE = 4.6 ms, flip angle = 8°, FOV = 240 mm, Matrix = 256 × 256 × 160 and slice thickness = 1 mm.

#### Data Processing

Statistical parametric software (SPM8, Welcome Trust Center for Neuroimaging, http://www.fil.ion.ucl.ac.uk/spm/software/spm8) was used to analyze the data with default parameters. All images were partitioned into gray and white matter (WM) and cerebrospinal fluid (CSF). Images were bias-corrected, tissue classified, and registered using linear (12-parameter affine) and non-linear transformations (warping), within a unified model (Ashburner and Friston, 2005). Subsequently, analyses were performed on the volume of the GM segments, which were multiplied by the non-linear components derived from the normalization matrix in order to preserve actual GM values locally. The resulting GM images were smoothed with a Gaussian kernel of 8 mm full width at half maximum (FWHM). Anatomical labeling of significant clusters was done by means of the anatomical automatic labeling toolbox (AAL; Tzourio-Mazoyer et al., 2002).

#### Statistical Analyses

For the statistical analysis, we excluded all voxels with a GM value below 0.1 (maximum value: 1) to avoid possible edge effects around the border between gray and WM and to include only voxels with sufficient GM proportion. To answer our specific questions, we performed a full factorial model to compare the two groups (HS/LE individuals versus HE/LS individuals; using probably contrasts 1 -1 and -1 1) to look for GM changes include age and head size as covariates. Head size were measured semi-automatically using MIDAS (Freeborough et al., 1997) using the total intracranial volume measurement as described by Whitwell et al. (2001). We applied FWE correction and report statistical significance at *p* < 0.05.

## Results

**FIGURE 1:**
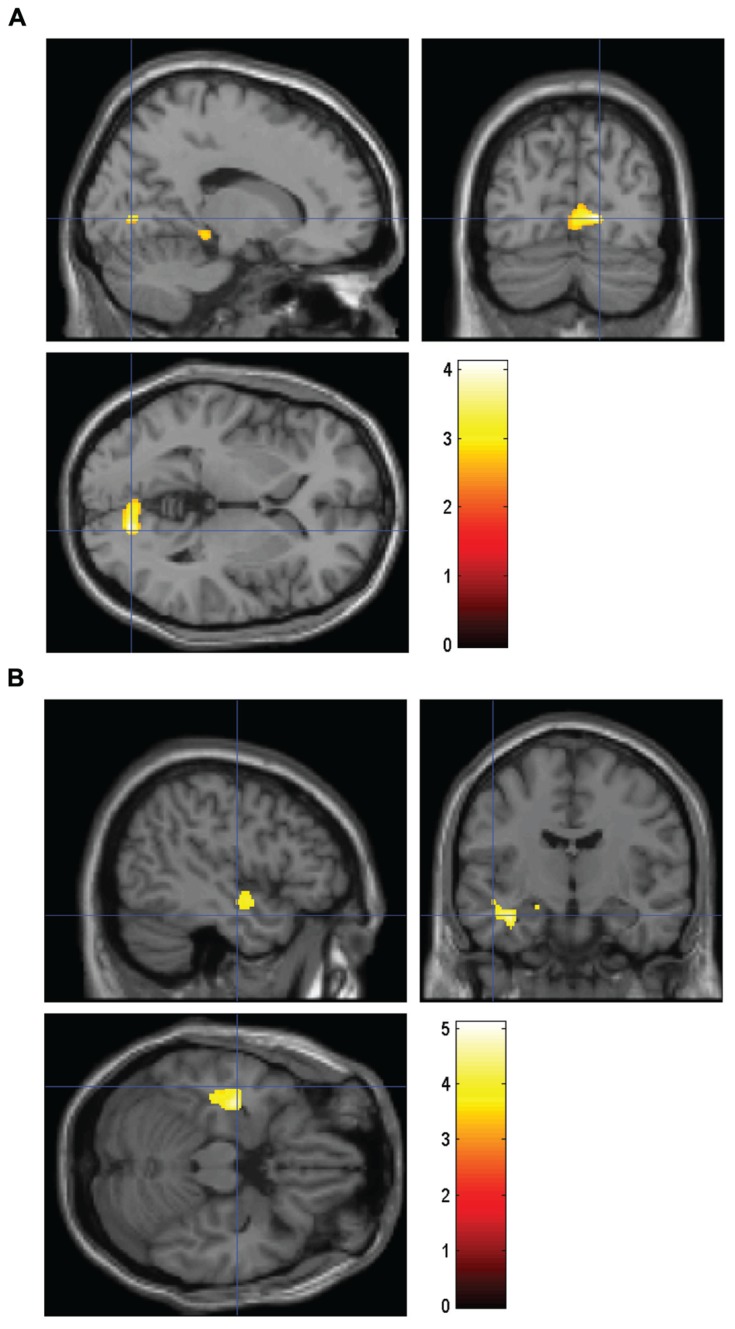
**Volume comparing HS/LE participants to HE/LS participants. (A)** Regions of relatively greater volume in HS/LE compared to HE/LS projected onto a T1-weighted image for the right parahippocampus (14 -75 1), the right hippocampus (11 -4 -15), the right cuneus (8 -79 18), and the left orbitofrontal cortex (-15 9 -20). **(B)** Regions of relatively decreased volume in HS/LE compared to HE/LS projected onto a T1-weighted image for the left inferior temporal cortex (-41 -16 -17), the left insula (-48 -4 -11), and the left amygdala (-18 -15 -14). The color bar represents the *t*-score.

The brain analysis of modulated data shows an increase in GM volume in the parahippocampus, the hippocampus, the cuneus and the orbitofrontal cortex, and a decrease in GM volume in the inferior temporal cortex, the insula and the amygdala in the HS/LE participants relative to the HE/LS participants (see **Table 2** and **Figure 1** for a detailed overview).

**Table 2:** Local maxima from the different contrasts highlighting gray matter (GM) difference between groups, obtained using VBM analysis.

	Side	Coordinates			*T*	Cluster size (*k*)
			MNI			
**Modulated**						
HS/LE group= SQ > EQ						
Parahippocampus	R	14	-75	1	4.10	477
Hippocampus	R	11	-4	-15	3.17	172
Cuneus	R	8	-79	18	2.88	46
Orbitofrontal cortex	L	-15	9	-20	2.80	38
HE/LS group= EQ < SQ						
Inferior temporal cortex	L	-41	-16	-17	3.16	735
Insula	L	-48	-4	-11	2.88	
Amygdala	L	-18	-15	-14	2.73	68

*L, left; R, Right.*

## Discussion

As mentioned in the introduction, GM structure abnormalities have been consistently identified in ASD in various social brain areas (Pelphrey et al., 2011). Some of these brain areas are also implicated in our study. Specifically, we find increased GM volume clusters in the parahippocampus, hippocampus, cuneus and orbitofrontal cortex, and decreased GM volume clusters in the inferior temporal cortex, insula, and amygdala in the HS/LE individuals relative to the HE/LS individuals.

Social functions, which are typically impaired in individuals with ASD, appear to correlate with function in the frontal lobe (the orbitofrontal cortex, insula, and medial prefrontal cortex), the temporal cortex and the amgydala, which are part of the social brain network. A variety of the social brain network regions show increases or decreases in GM in individuals with ASD (Pelphrey et al., 2011), and in our HS/LE individuals relative to the HE/LS individuals.

### Temporal–Occipital Area

Our study found GM volume increases in the occipital-temporal regions in the HS/LE group relative to the HE/LS group in the right cuneus (adjacent to the lingual gyrus), right hippocampus, and right parahippocampus. The meta-analysis by Nickl-Jockschat et al. (2012) found structural abnormalities in GM and WM in the right medial temporal lobe (inferior fronto-occipital fascicle, inferior longitudinal fascicle, and hippocampus) in individuals with ASD, and Ecker et al. (2013) found abnormal GM volume clusters in the lateral occipital lobe in individuals with autism.

The cuneus is part of the occipital lobe, projects to the lingual gyrus, plays a role in visual processing and is known to respond to reward, anticipatory, attention, and working memory manipulations. Sabbagh et al. (2009) hypothesize that differential activation in the cuneus may reflect the use of “mental imagery” (1158) during mindreading, or the extent to which mindreading relies on “reflecting upon one’s own knowledge states” (1157). The lingual gyrus is known to be involved in the encoding and recollection of visual memories (Machielsen et al., 2000), as well as selective attention (Mangun et al., 1998). Several studies have shown that visual processing differs in individuals with ASD compared to healthy controls (Lahaie et al., 2006; Pitcher et al., 2011; Arcurio et al., 2012).

Our study finds increased left hippocampal GM volume in the HS/LE group relative to the HE/LS group. Rojas et al. (2006) report increased GM in the medial temporal lobe area in the left hippocampus, left middle temporal, and right fusiform gyrus in individuals with ASD compared to normal controls. Schumann et al. (2004) report bilaterally enlarged hippocampal volume in children and adolescents with ASD compared to normal controls. Although some inconsistent findings have been reported regarding volumetric abnormalities in the hippocampus of individuals with ASD (e.g., see Palmen et al., 2006; Zeegers et al., 2008 which did not find disturbances in the hippocampus), the meta-analysis by Nickl-Jockschat et al. (2012) finds consistent abnormalities in the hippocampus of individuals with ASD. The hippocampus is part of the medial temporal lobe and is known to be involved in unconscious relational memory encoding and autobiographical memory (Tanweer et al., 2010; Duss et al., 2014).

Moreover, Ring et al. (1999) found greater activation of the right ventral occipital-temporal regions in individuals with autism during an embedded figures test (i.e., a test of local processing and visual search strategy) and concluded that individuals with autism depend “to an abnormally large extent on visual systems for object analysis” (1305). As mentioned, greater GM volume in our HS/LE individuals relative to our HE/LS individuals is found in this region in our study.

Interestingly, although individuals with ASD are found to have enhanced visual processing skills, these individuals are also found to have reduced face-based *mindreading* skills (i.e., identifying complex cognitive-emotional states from faces or face parts). A previous fMRI study found that the HS/LS individuals who participated in this study performed comparably accurate, but significantly slower on the ‘revised reading the mind in the eyes’ test compared to the HE/LS individuals (Focquaert et al., 2010). The test is a validated advanced theory of mind test involving complex mental states (e.g., social emotions). It is designed to check how well participants can ‘tune in’ to others’ thoughts and emotions (Baron-Cohen, 2003).

Our study finds reduced GM volume clusters in the left inferior temporal cortex in the HS/LE group relative to the HE/LS group. Abnormal fusiform face area activation when viewing faces in individuals with ASD has been reported in some studies (e.g., Critchley et al., 2000; Schultz et al., 2000), although these results have been contested (Hadjikhani et al., 2006). Overall, several functional neuroimaging studies have shown that atypical temporal lobe functioning is implicated in abnormal face recognition and emotional face-based mindreading in individuals with ASD (Baron-Cohen et al., 1999; Critchley et al., 2000; Schultz et al., 2000; Pierce et al., 2004; Dalton et al., 2005).

### Amygdala

The amygdala is a key social brain area occupying a central place in ASD research. Our study finds decreased GM volume in the left amygdala in the HS/LE individuals relative to the HE/LS individuals. Functional and volumetric amygdala abnormalities have been consistently found in ASD, although some heterogeneity exists in volumetric findings (Bellani et al., 2013). Enlargement of the amygdala is typically found in children (Schumann et al., 2004). Most studies suggest that the amygdala is smaller in adults with ASD (e.g., Aylward et al., 1999; Nacewicz et al., 2006), although preserved size has been reported in some studies (e.g., Palmen et al., 2006). Also, the meta-analysis by Nickl-Jockschat et al. (2012) finds consistent GM decreases in the left hippocampus/amygdala in individuals with ASD compared to controls.

The amygdala plays a role in the recognition of the emotional states of others through analysis of their facial expressions (e.g., Morris et al., 1996) and in the experience and regulation of emotion overall (LeDoux, 2000). A recent volumetric study comparing ASD to schizophrenia found that mindreading abilities are significantly correlated with amygdala volume in the individuals with ASD (Radeloff et al., 2014). It has been argued that individuals with high-functioning ASD show a neuropsychological profile that is characteristic of amygdala damage involving selective impairment of face-based mindreading (e.g., difficulty attributing fear, perceiving eye-gaze direction and remembering faces) due to the developmental abnormalities of the amygdala (Howard et al., 2000).

### Orbitofrontal Cortex

The orbitofrontal cortex is a central part of the social brain network (Pelphrey et al., 2011). Our study finds increased GM volume in the orbitofrontal cortex in the HS/LE individuals relative to the HE/LS individuals. Hyde et al. (2010) report GM increases in the orbitofrontal area of individuals with ASD and Salmond et al. (2003) previously reported orbitofrontal volumetric abnormalities in adolescents with ASD. As mentioned, abnormalities in the orbitofrontal cortex may be linked to social behavior difficulties (e.g., difficulties when making social judgments, impaired empathy) in individuals with ASD and possibly in individuals that score high in autistic traits. The orbitofrontal cortex has also been implicated in repetitive behaviors in several studies (Hyde et al., 2010).

### Insula

The insula is, similar to the other volumetric regions we identified, part of the social brain network and plays a role in experiential affective and empathic processing. Research suggests that the insula is “part of a ‘salience network’ integrating external sensory stimuli with internal states” (Uddin and Menon, 2009, p. 1198). Our study finds smaller GM volume in the left insula in the HS/LE individuals relative to the HE/LS individuals. A recent large-scale connectivity and volumetric study identified the left insula as one of two common loci of dysfunction in ASD (DiMartino et al., 2014). Moreover a recent volumetric study (Radeloff et al., 2014) comparing individuals with ASD to individuals with schizophrenia, whom both suffer from abnormalities in the social brain areas, found smaller GM volume in the left insula, and bilateral amygdala, and higher GM volume in the occipital medial area in the ASD individuals.

As mentioned in the introduction, the recent DSM-V debate focused on psychiatric disorders as dimensional rather than categorical entities, and heterogeneous in nature rather than representing discrete disease entities (Frances, 2009). Based on accumulating evidence that places autistic traits on a continuum in the general population, with clinical ASD representing the extreme end of this continuous distribution (Whitehouse et al., 2011), it is possible that the difference between individuals having the diagnosis of ASD and individuals with high autistic traits reflects a matter of degree in terms of atypical structural and functional brain results. An interesting further path of research would be to focus on those factors that may differentiate between individuals with ASD and HS/LE individuals in terms of brain structure and functioning.

#### Limitations

Although one might look for individuals with subclinical manifestations of ASD (using a clinical diagnostic measure), we aim to investigate autistic traits that manifest themselves in a continuous manner in the population at large and that are not exclusively linked to specific diagnostic criteria. By not necessarily restricting our findings to one particular clinical disorder, our research may potentially bear wider importance to our understanding of psychiatry and impaired psychiatric functioning in general. Moreover, this approach is in line with current findings of co-morbidity in psychiatry and the heterogeneous nature of the existing ASD diagnosis.

One limitation of the current study might be the low number of participants. However, one has to take into account that participants were at the end of the spectrum. That is, participants need to be healthy, not been diagnosed with Asperger syndrome and score high on systemizing and low on empathizing or the opposite scoring low to average on systemizing and high to average on empathizing. As mentioned in the participants section, the HS/LE group scored, on average, within the Asperger range on both systemizing quotient and empathizing quotient, whereas the HE/LS group scored, on average, higher than most normal men on the empathizing quotient and lower than most men on the systemizing quotient. In addition, participants were matched for gender, age, and needed to be right-handed.

## Conclusion

In view of the new DSM-5 ASD diagnosis in which the autism spectrum reflects a group of neurodevelopmental disorders existing on a continuum from mild to severe expression of autistic traits, and recent empirical findings showing a continuous distribution of autistic traits in the general population, our voxel based morphometry study compares normal individuals with high autistic traits in terms of systemizing and empathizing to normal individuals with low autistic traits in terms of empathizing and systemizing. Several brain regions showing structural differences in our individuals with high autistic traits in terms of empathizing and systemizing (HS/LE group) relative to our HE/LS individuals, show overlap with brain regions specifically linked to ASD in previous studies. Specifically, we find overlapping neuroanatomical clusters in a variety of social brain areas such as the temporo-occipital area (including the cuneus, hippocampus, and parahippocampus), the inferior temporal cortex, the orbitofrontal cortex, the insula, and the amygdala.

## Conflict of Interest Statement

The authors declare that the research was conducted in the absence of any commercial or financial relationships that could be construed as a potential conflict of interest.
